# Quantum Fisher information of atomic system interacting with a single cavity mode in the presence of Kerr medium

**DOI:** 10.1038/s41598-019-39183-5

**Published:** 2019-02-25

**Authors:** N. Zidan, H. F. Abdel-Hameed, N. Metwally

**Affiliations:** 1 Mathematics Department, Faculty of Science, Sohag University, Sohag, Egypt; 2 Mathematics Department, College of Science, Jouf University, Sakaka, Saudi Arabia; 3 Mathematics Department, Khurma University College, Taif University, Al-Taif, Saudi Arabia; 4 Mathematics Department, College of Science, Bahrain University, Zallaq, Bahrain; 5 Mathematics Department, Faculty of Science, Aswan University, Aswan, Egypt

## Abstract

The quantum Fisher information of an atomic system interacting with a single cavity mode in the presence of Kerr medium is discussed. It is shown that quantum Fisher information for an initial separable atomic system is larger than that depicted for the initial entangled atomic system. For initial vacuum state of the cavity mode, the quantum Fisher information with respect to the Kerr medium and the phase decoherence parameter is larger than that displayed for the detuning parameter. Both phase decoherence and Kerr medium have the same effect on the decay of quantum Fisher information, while they have an opposite effect on its maximum values.

## Introduction

Fisher information is proposed to detect the precision of the parameter in estimation theory^1^. Extending the estimation theory from the classical regime to the quantum regime, quantum Fisher information plays a paramount role in quantum information theory^2–4^ and quantum metrology^5,6^. Quantum Fisher information has been widely studied theoretically and experimentally^7–18^. Quantum Fisher information has become a highly studied subject because it measures the phase sensitivity of the systems^19–23^. Moreover, quantum Fisher information in non-inertial frames has been investigated for different systems, such as the performance of quantum Fisher information under the Unruh-Hawking effect in the context of relativistic quantum information theory^24^, the dynamic of the teleported quantum Fisher information^25^, and the Unruh acceleration effect on the precision of parameter estimation for a general two-qubit system^26^. The existence of the maximal quantum information matrix in multi-parameter quantum estimation^27^ and entanglement detection have been discussed extensively^28,29^. The dynamics of quantum Fisher information in the two-level system coupled to a single bosonic reservoir^30,31^ and multiple bosonic reservoirs^32,33^ has been discussed. Quantum Fisher information is used as a predictor of decoherence in the preparation of spin-cat states for quantum metrology^34^. Also, quantum Fisher information of a single qubit has quantified^35,36^. The dynamics of quantum Fisher information is studied and emplayed as a measure to quantify the precision of the estimation of two interacting qubits subject to decoherence^37^. The weak measurement based pre- and post- flips is used to protect the average quantum Fisher information in the independent amplitude damping channel for N-qubit GHZ states^38^. The energy-level crossing behavior in a two-dimensional well with the Rashba and Dresselhaus-orbit coupling is studied and the approximate ground state and its quantum Fisher information via performing a unitary transformation is obtained^39^ Furthermore, enhancing parameter precision optimal quantum estimation by direct quantum feedback^40^ and quantum screening were reported^41^. The studying how to estimate two parameters in a spin-boson dephasing system by periodical projective measurements has been investigated^42^. The quantum Fisher information of a two charged qubits system interact locally with a dephasing channel is quantified with respect to the charged qubits and the channel parameters^43^. For the Jaynes-Cummings-Dicke model, enhanced spin squeezing and quantum entanglement have been explained^44^. Also, it has been shown that the Kerr-like medium plays an important role in generating twin beams by parametric amplification four-wave-mixing beams and triplet beams from the parametric amplification six-wave mixing process, where it can be used as a control parameter^45^. Therefore we are motivated to consider, an atomic system containing two-atoms interacting with a single cavity mode with Kerr medium in the presence of a phase decoherence. Our contribution focus on estimating the parameters of the system by evaluating the quantum Fisher information. We focus on three parameters, the Kerr medium (*χ*), phase decoherence (*γ*) and detuning (Δ) and study the effect of these parameters on the behavior of quantum Fisher information. As an application of this model is generating a three qubit entangled state between the two atoms and the cavity mode. Also, this system may be used to teleport information from one party to another one between the three qubits. Moreover, it can be used to perform entanglement swapping between the one atom of the atomic system and the cavity mode. Therefore, It is important to know the values of the parameters that maximize/minimize the efficiency to perform these applications.

This paper is organized as follows: We introduce the model and calculation the reduced density matrix of two-two level atoms in sect. 2. In sect. 3, some properties of the quantum Fisher information are given and then we estimate the parameters of the system. Numerical discussion of our results is presented in sect. 4. Finally, the conclusion is given in sect. 5.

## The Suggested Model

It is assumed that an atomic system consists of two two-level atoms interacting with a single cavity mode field which is initially prepared in the Fock state $|n+1\rangle$. In the rotating wave approximation, the Hamiltonian which describes this system is given by^46^:

1 $$ {\mathcal H} ={\omega }_{f}{a}^{\dagger }a+\frac{{\omega }_{a}}{2}\sum _{j=A,B}\,{\sigma }_{z}^{(j)}+\kappa \sum _{j=A,B}\,\{a{\sigma }_{+}^{(j)}+{a}^{\dagger }{\sigma }_{-}^{(j)}\}+\chi ({a}^{\dagger }a{a}^{\dagger }a),$$

where $a({a}^{\dagger })$ denotes to the annihilation (creation) operator of the single cavity mode, *ω*_*a*_ and *ω*_*f*_ are the atomic and field transition frequencies, respectively. Here *χ* represents the coupling of the field induced by the Kerr medium and *κ* is the coupling constant between the atoms and field. ${\sigma }_{z}^{(j)}=\mathrm{|1}{\rangle }_{j}{\langle \mathrm{0|}-\mathrm{|0}\rangle }_{j}\langle \mathrm{1|,}$ ${\sigma }_{+}^{(j)}=\mathrm{|1}{\rangle }_{j}\langle \mathrm{0|,}$ ${\sigma }_{-}^{(j)}=\mathrm{|0}{\rangle }_{j}\langle \mathrm{1|}$ with |1〉_*j*_ and |0〉_*j*_ being the excited and ground states of *jth* atom (*j* = *A*, *B*). By the basis, |*n*, 11〉, |*n* + 1, 10〉, |*n*+1, 01〉 and |*n* + 2,00〉 the eigenvalues of Hamiltonian (1) can be given by:

2 $$\begin{array}{rcl}{E}_{1} & = & {\rm{\Delta }}-\mathrm{2(}n+\mathrm{1)}\chi ,\,{E}_{2}=-\,\chi ,\\ {E}_{\mathrm{3,4}} & = & \frac{1}{2}({\rm{\Delta }}-\mathrm{(2}n+\mathrm{3)}\chi \mp \delta ),\end{array}$$

where $\delta =\sqrt{8{\kappa }^{2}\mathrm{(2}n+\mathrm{3)}+{(-{\rm{\Delta }}+\chi +2n\chi )}^{2}}$ and ${\rm{\Delta }}={\omega }_{a}-{\omega }_{f}$ is the detuning of the cavity field. The corresponding eigenvectors are given by:

3 $$\begin{array}{rcl}|{\psi }_{1}\rangle  & = & -\sqrt{\frac{n+2}{n+3}}|n\mathrm{,11}\rangle +\sqrt{\frac{n+1}{n+3}}|n+\mathrm{2,00}\rangle ,\\ |{\psi }_{2}\rangle  & = & -\,\frac{1}{\sqrt{2}}(-\,|n+\mathrm{1,}\,10\rangle +|n+\mathrm{1,}\,01\rangle ),\\ |{\psi }_{3}\rangle  & = & \frac{2\kappa \sqrt{n+1}}{\sqrt{{\eta }_{1}}}|n\mathrm{,11}\rangle +\frac{2\kappa \sqrt{n+2}}{\sqrt{{\eta }_{1}}}|n+\mathrm{2,00}\rangle \\  &  & -\frac{\sqrt{{\eta }_{1}}}{2\delta }(|n+\mathrm{1,10}\rangle +|n+\mathrm{1,01}\rangle ),\,\\ |{\psi }_{4}\rangle  & = & \frac{2\kappa \sqrt{n+1}}{\sqrt{{\eta }_{2}}}|n\mathrm{,11}\rangle +\frac{2\kappa \sqrt{n+2}}{\sqrt{{\eta }_{2}}}|n+\mathrm{2,00}\rangle \\  &  & +\,\frac{\sqrt{{\eta }_{2}}}{2\delta }(|n+\mathrm{1,10}\rangle +|n+\mathrm{1,01}\rangle )\mathrm{.}\end{array}$$

Here $${\eta }_{1}=\delta (\delta +{\rm{\Delta }}-\mathrm{(2}n+\mathrm{1)}\chi )$$, $${\eta }_{2}=\delta (\delta -{\rm{\Delta }}+\mathrm{(2}n+\mathrm{1)}\chi )$$.

The master equation which governs the time evolution of the system under the Markovian approximation is given by^47^:

4 $$\frac{d\rho }{dt}=-\,i[ {\mathcal H} ,\,\rho ]-\frac{\gamma }{2}[ {\mathcal H} ,[ {\mathcal H} ,\,\rho ]],$$

where *γ* is the phase decoherence coefficient. The formal solution of the master Eq. (4) may be expressed in the following form^48^:

5 $$\rho (t)=\sum _{k\mathrm{=0}}^{\infty }\,\frac{{(\gamma t)}^{k}}{k!}{ {\mathcal M} }^{k}(t)\rho \mathrm{(0)}{ {\mathcal M} }^{\dagger k}(t),$$

where *ρ*(0) is the density operator of the initial atom-field system. ${ {\mathcal M} }^{k}(t)$ is defined as:

6 $${ {\mathcal M} }^{k}(t)={ {\mathcal H} }^{k}\exp (-it {\mathcal H} )\exp (-\frac{\gamma t}{2}{ {\mathcal H} }^{2}).$$

Let us assume that the cavity field is initially prepared in the Fock state $|{\psi }_{f}\mathrm{(0)}\rangle =|n+1\rangle$ and the atomic system is initially prepared in the entangled states $|{\psi }_{AB}\mathrm{(0)}\rangle =\,\cos \,\phi \mathrm{|10}\rangle +\,\sin \,\phi \mathrm{|10}\rangle$. Then the initial state of the total system may be written as:

7 $$|{\psi }_{s}\mathrm{(0)}\rangle =|{\psi }_{AB}\mathrm{(0)}\rangle \otimes |{\psi }_{f}\mathrm{(0)}\rangle \mathrm{.}$$

Using Eqs (5) and (7) we can obtain the reduced density matrix of the atomic system *ρ*_*AB*_(*t*) after taking the trace over the field in the form

8 $$\begin{array}{rcl}{\rho }_{AB}(t) & = & {\rho }_{11}\mathrm{|11}\rangle \langle \mathrm{11|}+{\rho }_{22}\mathrm{|10}\rangle \langle \mathrm{10|}+{\rho }_{33}\mathrm{|01}\rangle \langle \mathrm{01|}\\  &  & +\,{\rho }_{23}\mathrm{|10}\rangle \langle \mathrm{01|}+{\rho }_{32}\mathrm{|01}\rangle \langle \mathrm{10|}+{\rho }_{44}\mathrm{|00}\rangle \langle \mathrm{00|,}\end{array}$$

where

$$\begin{array}{rcl}{\rho }_{11} & = & \frac{2{\kappa }^{2}(n+\mathrm{1)}}{{\delta }^{2}}X,\,{\rho }_{44}=\frac{2{\kappa }^{2}(n+\mathrm{2)}}{{\delta }^{2}}X,\\ {\rho }_{22} & = & \frac{1}{2}-\frac{{\kappa }^{2}\mathrm{(2}n+\mathrm{3)}}{{\delta }^{2}}X\\  &  & +\,\frac{\cos (2\phi )}{4{\delta }^{2}}({Y}_{1}\,\cos [t(\frac{{\eta }_{1}}{2\delta })]+{Y}_{2}\,\cos [t(\frac{{\eta }_{2}}{2\delta })]),\\ {\rho }_{33} & = & \frac{1}{2}-\frac{{\kappa }^{2}\mathrm{(2}n+\mathrm{3)}}{{\delta }^{2}}X\\  &  & -\frac{\cos (2\phi )}{4{\delta }^{2}}({Y}_{1}\,\cos [t(\frac{{\eta }_{1}}{2\delta })]+{Y}_{2}\,\cos [t(\frac{{\eta }_{2}}{2\delta })]),\\ {\rho }_{23} & = & {\rho }_{32}^{\ast }=\frac{\sin (2\phi )}{2}-\frac{{\kappa }^{2}\mathrm{(2}n+\mathrm{3)}}{{\delta }^{2}}X\\  &  & -\frac{i\,\cos (2\phi )}{4{\delta }^{2}}({Y}_{1}\,\sin [t(\frac{{\eta }_{1}}{2\delta })]-{Y}_{2}\,\sin [t(\frac{{\eta }_{2}}{2\delta })])\mathrm{.}\end{array}$$

with

$$\begin{array}{rcl}X & = & (1+\,\sin \,\mathrm{(2}\phi ))(1-\exp [-\frac{\gamma t{\delta }^{2}}{2}])\cos (t\delta ),\\ {Y}_{1} & = & {\eta }_{1}\,\exp [-\,\frac{\gamma t}{2}{(\frac{{\eta }_{1}}{2\delta })}^{2}],\,{Y}_{2}={\eta }_{2}\,\exp [-\,\frac{\gamma t}{2}{(\frac{{\eta }_{2}}{2\delta })}^{2}]\end{array}$$

## Quantum Fisher Information

Quantum Fisher information of any parameter characterizes the sensitivity of the state with respect to changes of this parameter. Let us assume that the parameter *τ* is coded in the quantum state *ρ*_*τ*_. The quantum Fisher information with respect to the parameter *τ* is defined as^2,49^:

9 $${ {\mathcal F} }_{\tau }=Tr({\rho }_{\tau }{\pounds }_{\tau }^{2})=Tr(\frac{\partial {\rho }_{\tau }}{\partial \tau }{\pounds }_{\tau }),$$

where ${\pounds }_{\tau }$ is so-called symmetric logarithmic derivative which is defined by:

10 $$\frac{\partial {\rho }_{\tau }}{\partial \tau }=({\pounds }_{\tau }{\rho }_{\tau }+{\rho }_{\tau }{\pounds }_{\tau })\mathrm{/2.}$$

Typically there are three methods to calculate the quantum Fisher information^24^. The most frequently method used is the diagonalizing of the matrix ${\rho }_{\tau }=\sum _{i\mathrm{=1}}^{n}{\lambda }_{i}|{V}_{i}\rangle \langle {V}_{i}|$, where *λ*_*i*_ and $|{V}_{i}\rangle$ are the eigenvalues and eigenvectors of the density operator *ρ*_*τ*_, respectively. Then the quantum Fisher information ${ {\mathcal F} }_{\tau }$ with respect to the parameter τ is written as^50,51^:

11 $$\begin{array}{rcl}{ {\mathcal F} }_{\tau } & = & \sum _{i\mathrm{=1}}^{n}\,\frac{1}{{\lambda }_{i}}{(\frac{\partial {\lambda }_{i}}{\partial \tau })}^{2}+4\sum _{i\mathrm{=1}}^{n}\,{\lambda }_{i}(\langle \frac{\partial {V}_{i}}{\partial \tau }|\frac{\partial {V}_{i}}{\partial \tau }\rangle -{|\langle {V}_{i}|\frac{\partial {V}_{i}}{\partial \tau }\rangle |}^{2})\\  &  & -\,8\sum _{i\ne j}^{n}\,\frac{{\lambda }_{i}{\lambda }_{j}}{{\lambda }_{i}+{\lambda }_{j}}{|\langle {V}_{i}|\frac{\partial {V}_{j}}{\partial \tau }\rangle |}^{2}.\end{array}$$

To calculate the quantum Fisher information of the suggested atomic system, one needs to find the eigenvalues and eigenvectors of the reduced density operator Eq. (8) of the atomic system. In this context, the eigenvalues are given explicitly by:

12 $${\lambda }_{1}={\rho }_{11},\,{\lambda }_{2}={\rho }_{44},\,{\lambda }_{\mathrm{3,4}}=\frac{1}{2}({\rho }_{22}+{\rho }_{33}\pm \sqrt{{({\rho }_{22}-{\rho }_{33})}^{2}+4{|{\rho }_{23}|}^{2}}),$$

with the corresponding eigenvectors:

13 $$\begin{array}{rcl}|{V}_{1}\rangle  & = & \mathrm{(1,}\,\mathrm{0,}\,\mathrm{0,}\,\mathrm{0),}\,|{V}_{2}\rangle =\mathrm{(0,}\,\mathrm{0,}\,\mathrm{0,}\,\mathrm{1),}\\ |{V}_{3}\rangle  & = & \frac{1}{|{\rho }_{23}|\sqrt{{|{\rho }_{23}|}^{2}+{({\lambda }_{3}-{\rho }_{33})}^{2}}}\mathrm{(0},\,({\lambda }_{3}-{\rho }_{33}){\rho }_{23},\,{|{\rho }_{23}|}^{2},\,\mathrm{0),}\\ |{V}_{4}\rangle  & = & \frac{1}{|{\rho }_{23}|\sqrt{{|{\rho }_{23}|}^{2}+{({\lambda }_{4}-{\rho }_{33})}^{2}}}\mathrm{(0,}\,({\lambda }_{4}-{\rho }_{33}){\rho }_{23},\,{|{\rho }_{23}|}^{2},\,\mathrm{0).}\end{array}$$

Making use of Eq. (11), we can obtain the quantum Fisher information of the density matrix *ρ*_*AB*_(*t*) given in Eq. (8). As its expression is very complicated, we will display the results mainly by numerical simulations in the following section.

## Numerical Discussion

### Quantum Fisher information with respect to the Kerr medium

In this subsection, we estimate the parameter *χ* by evaluating the corresponding quantum Fisher information, namely ${ {\mathcal F} }_{\chi }$. The effect of the phase decoherence parameter *γ*, the mean photon number *n*, the detuning parameter Δ and the Kerr medium parameter *χ* on the quantum Fisher information ${ {\mathcal F} }_{\chi }$ will be discussed.

**Figure 1:**
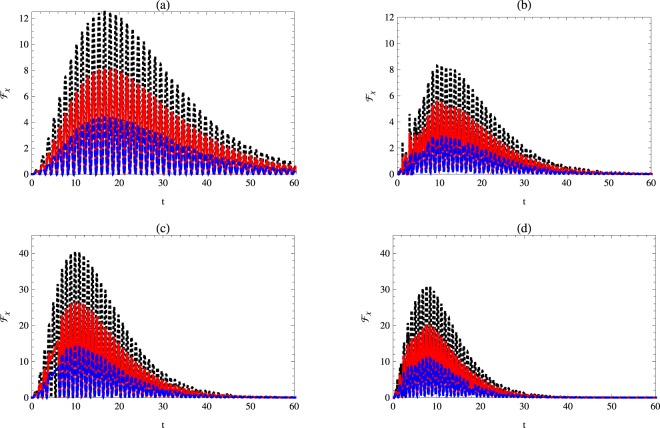
Quantum Fisher information ${ {\mathcal F} }_{\chi }$ as a function of time *t* with *κ* = 1, *γ* = 0.02 and *χ* = 0.1 for different initial state settings. The dotted-black, solid-red, and dashed-blue curves represent ${ {\mathcal F} }_{\chi }$ for a weight angle $\theta =\mathrm{0,}\,\pi \mathrm{/8,}$ and *π*/3, respectively. Here: (**a**) *n* = 0, Δ = 0, (**b**) *n* = 0, Δ = 1 (**c**) *n* = 1, Δ = 0, (**d**) *n* = 1, Δ = 1.

Figure 1 describes the quantum Fisher information behavior with respect to the Kerr-like medium parameter for different cases. It is assumed that the atomic-field system is initially prepared in the resonance and non-resonance cases. The field is prepared in the vacuum and one photon Fock states, while the atomic system is prepared in different initial states; separable and partially entangled atomic states. As soon as the interaction is switched on ${ {\mathcal F} }_{\chi }$ increases suddenly to reach its maximum values and then oscillates between its upper and lower bounds. As the time increases the upper bounds decrease to vanish completely at the further time. Moreover, the upper and lower bounds of ${ {\mathcal F} }_{\chi }$ depend on the initial state settings of the atomic system, where the upper bounds of ${ {\mathcal F} }_{\chi }$ decrease as the weight angle increases. This shows that the possibility of estimating the *χ* parameter increases if the initial atomic system is coded to classical information. Additionally, the resonance and non-resonance cases have a clear effect. At the resonance case, the upper bounds of ${ {\mathcal F} }_{\chi }$ are larger than those displayed for the non-resonance case. The effect of photon number can be seen clearly when compare between Fig. 1(a) with 1(c) (or 1(b) with 1(d)) where the upper bounds of ${ {\mathcal F} }_{\chi }$ for one-photon are higher than that depicted for vacuum state. On the other hand, for the non-resonance case, the upper bounds of the ${ {\mathcal F} }_{\chi }$ are smaller than those are displayed for the resonance case as shown from Fig. 1(c,d).

**Figure 2:**
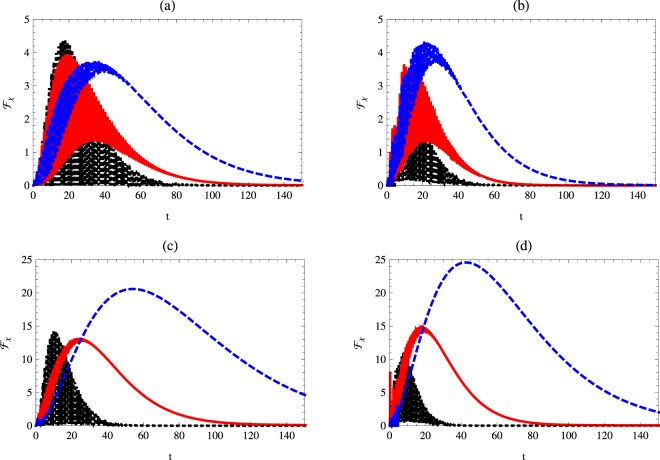
Quantum Fisher information ${ {\mathcal F} }_{\chi }$ as a function of time *t* with *κ* = 1, *γ* = 0.02 and $\theta =\pi \mathrm{/3}$ for different values of Kerr medium. The dotted-black, solid-red and dashed-blue curves represent ${ {\mathcal F} }_{\chi }$ at *χ* = 0.1, 1 and *χ* = 2, respectively. Here: (**a**) *n* = 0, Δ = 0, (**b**) *n* = 0, Δ = 1 (**c**) *n* = 1, Δ = 0, (**d**) *n* = 1, Δ = 1.

Figure 2 displays the effect of different initial values of the Kerr medium where the atomic system is prepared in a partial entangled state with a weight angle $\theta =\pi \mathrm{/3}$. It is clear that at small values of *χ* the upper bonds of ${ {\mathcal F} }_{\chi }$ are larger than those displayed for larger values of *χ*. On the other hand, for the further time, the quantum Fisher information decreases gradually to vanish completely at very high values of time *t*. The survival time of ${ {\mathcal F} }_{\chi }$ increases as *χ* increases. The resonance, non-resonance, vacuum and one photon cases have a noticeable effect on the upper bounds of ${ {\mathcal F} }_{\chi }$ and its survival time.

**Figure 3:**
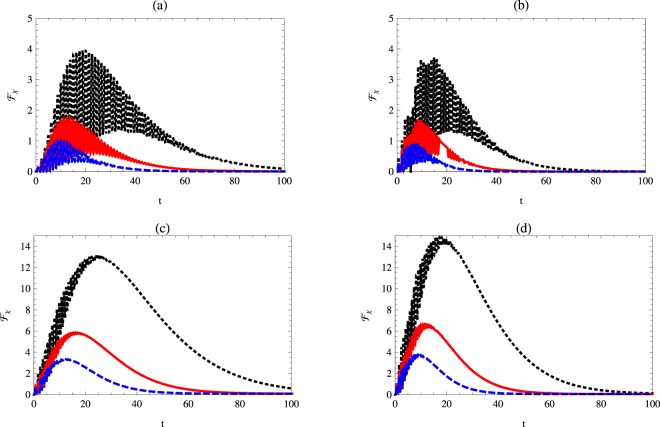
Quantum Fisher information ${ {\mathcal F} }_{\chi }$ as a function of time *t* with *κ* = 1 *χ* = 1 and $\theta =\pi \mathrm{/3}$ for different values of phase decoherence. The dotted-black, solid-red and dashed-blue curves represent ${ {\mathcal F} }_{\chi }$ at *γ* = 0.02, 0.03 and *γ* = 0.04, respectively. Here: (**a**) *n* = 0, Δ = 0, (**b**) *n* = 1, Δ = 1, (**c**) *n* = 1, Δ = 0, (**d**) *n* = 1, Δ = 1.

In Fig. 3 we investigate the effect of the phase decoherence parameter *γ* on the behavior of ${ {\mathcal F} }_{\chi }$. The general behavior shows that the quantum Fisher information decreases as the phase decoherence increases. The decay rate depends on the initial state of the cavity mode and the detuning between the atomic system and the cavity. It is clear that the upper bounds of ${ {\mathcal F} }_{\chi }$ are large for the one-photon and non-resonance case. From Figs 1–3 one may conclude that the precision of estimating the Kerr medium *χ* parameter depends on the initial state settings of the field and the atomic systems. The larger initial values of *χ*, the larger upper bounds of the quantum Fisher information ${ {\mathcal F} }_{\chi }$. Although the maximum value of ${ {\mathcal F} }_{\chi }$ decreases as the phase decoherence increases, one may increase these values by considering the detuning and the one-photon case.

### Quantum Fisher information with respect to the detuning

In this section, the quantum Fisher information with respect to the detuning parameter is discussed. It is assumed that the atomic system is initially prepared in a partial entangled state and the cavity is prepared in a vacuum and one photon case.

**Figure 4:**
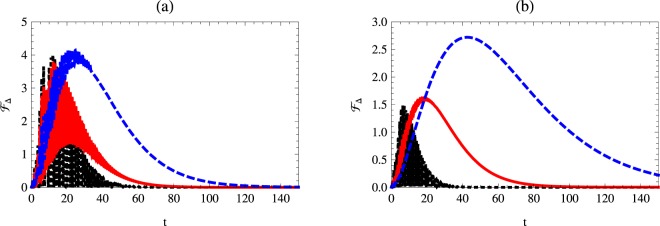
Quantum Fisher information ${ {\mathcal F} }_{{\rm{\Delta }}}$ as a function of time *t* with *κ* = 1, *γ* = 0.02 and $\theta =\pi \mathrm{/3}$ for different values of Kerr medium. The dotted-black, solid-red and dashed-blue curves represent ${ {\mathcal F} }_{\chi }$ at *χ* = 0.1, 1 and *χ* = 2, respectively. Here: (**a**) *n* = 0, Δ = 1, and (**b**) *n* = 1, Δ = 1.

**Figure 5:**
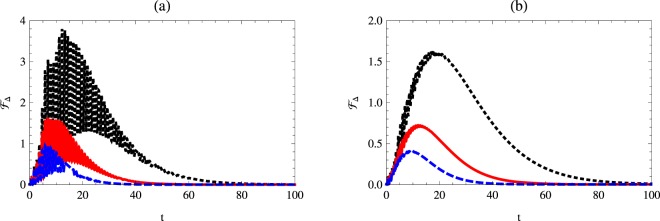
Quantum Fisher information ${ {\mathcal F} }_{{\rm{\Delta }}}$ as a function of time *t* with *κ* = 1, *χ* = 1 and $\theta =\pi \mathrm{/3}$ for different values of phase decoherence. The dotted-black, solid-red and dashed-blue curves represent ${ {\mathcal F} }_{\chi }$ at *γ* = 0.02, 0.03 and *γ* = 0.04, respectively. Here: (**a**) *n* = 0, Δ = 1, and (**b**) *n* = 1, Δ = 1.

Figure 4 shows the behavior of ${ {\mathcal F} }_{{\rm{\Delta }}}$ for different initial values of the *χ* parameter. The behavior is similar to that displayed in Fig. 2(a,b) but the maximum values of ${ {\mathcal F} }_{{\rm{\Delta }}}$ are smaller than those displayed for ${ {\mathcal F} }_{\chi }$. The one-photon cavity mode makes the oscillations of the quantum Fisher information ${ {\mathcal F} }_{{\rm{\Delta }}}$ collapse. Moreover, the survival time of ${ {\mathcal F} }_{{\rm{\Delta }}}$ is larger than that displayed for the vacuum case. The phase decoherence effect on the behavior of ${ {\mathcal F} }_{{\rm{\Delta }}}$ is described in Fig. 5, where different values of *γ* are considered. The general behavior is similar to that displayed for ${ {\mathcal F} }_{\chi }$ but has smaller maximum values.

### Quantum Fisher information with respect to the phase decoherence

**Figure 6:**
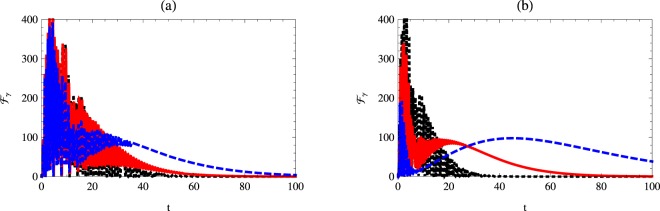
Quantum Fisher information ${ {\mathcal F} }_{\gamma }$ as a function of time *t* with *κ* = 1, *γ* = 0.02 and $\theta =\pi \mathrm{/3}$ for different values of Kerr medium. The dotted-black, solid-red and dashed-blue curves represent ${ {\mathcal F} }_{\chi }$ at *χ* = 0.1, 1 and *χ* = 2, respectively. Here: (**a**) *n* = 0, Δ = 1, and (**b**) *n* = 1, Δ = 1.

**Figure 7:**
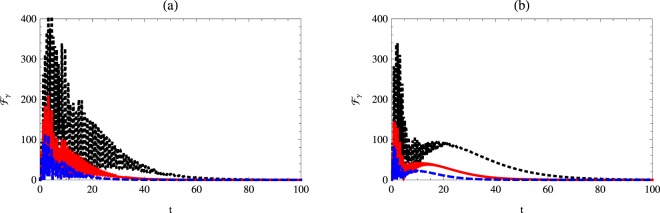
Quantum Fisher information ${ {\mathcal F} }_{\gamma }$ as a function of time *t* with *κ* = 1, *χ* = 1 and $\theta =\pi \mathrm{/3}$ for different values of phase decoherence. The dotted-black, solid-red and dashed-blue curves represent ${ {\mathcal F} }_{\chi }$ at *γ* = 0.02, 0.03 and *γ* = 0.04, respectively. Here: (**a**) *n* = 0, Δ = 1, and (**b**) *n* = 1, Δ = 1.

Quantum Fisher information with respect to ${ {\mathcal F} }_{\gamma }$ is described in Figs 6 and 7. The upper bounds of ${ {\mathcal F} }_{\gamma }$ are much larger than those displayed for ${ {\mathcal F} }_{\chi }$ and ${ {\mathcal F} }_{{\rm{\Delta }}}$.

As it is shown in Fig. 6 the effect of the Kerr medium on ${ {\mathcal F} }_{\gamma }$ shows that the larger values of *χ* make the upper bounds of ${ {\mathcal F} }_{\gamma }$ increase. The one-photon case of the cavity causes a delay of vanishing of the quantum Fisher information ${ {\mathcal F} }_{\gamma }$. The effect of the initial values of *γ* on ${ {\mathcal F} }_{\gamma }$ is displayed in Fig. 7.

The general behavior shows that ${ {\mathcal F} }_{\gamma }$ decays as *γ* increases. The decay rate depends on the initial state of the total system. At one photon cavity mode, the quantum Fisher information collapses and consequently, the minimum values are larger than those displayed at the vacuum state.

## Conclusion

In this contribution, a two-atom system interacting with a cavity mode which is initially prepared in the Fock state in presence of Kerr medium under decoherence is considered. The estimation of the Kerr medium, detuning, and the phase decoherence parameters are discussed in different initial states. Four different cases are considered (namely, resonance, non-resonance case, vacuum and one-photon cavity). First, it is important to mention that before the interaction is switched on, the Fisher information of all the estimated parameters is zero. Because, these parameters don’t appear on the atomic system before the interaction. However, it is shown that, quantum Fisher information is much larger than those depicted for the Kerr-medium and the detuning parameters. Also preparing the cavity mode in non-vacuum state causes an increase of the upper bounds of quantum Fisher information and its survival time too. The initial states settings of the atomic and the field systems play an important role in the behavior of the three kinds of the quantum Fisher information. Quantum Fisher information for the separable atomic system is larger than that displayed for entangling atomic system. The non-vacuum state of the field causes an increase the maximum values of quantum Fisher information with respect to the Kerr medium and the phase decoherence parameter. The Kerr medium and the phase decoherence have a similar effect on all the three types of the quantum Fisher information.
